# Peer factors and prosocial behavior among Chinese adolescents from difficult families

**DOI:** 10.1038/s41598-023-50292-0

**Published:** 2024-01-08

**Authors:** Yuexuan Mu, Benfeng Du

**Affiliations:** 1 School of Public Health, Soochow University, Suzhou, China; 2 Interdisciplinary Innovation Platform of Public Health and Disease Prevention and Control for Health Policy, Renmin University of China, Beijing, China

**Keywords:** Psychology, Risk factors

## Abstract

Adolescents from difficult families (ADF) is a vulnerable group in China, and there have been few studies focused on them at present. To improve the welfare system for vulnerable groups and gain a better understanding of the situation regarding ADF, it is important to identify the association between peer factors, family functioning, and prosocial behavior among ADF. 1047 adolescents aged 10–15 from difficult families were selected from 21 counties in 7 provinces across China based on the multistage stratified sampling method. Regression analysis and moderation analyses were performed to identify the association of prosocial behavior with peer factors and family functioning. Lower peer quality and poorer family functioning were significantly associated with less prosocial behavior. The was no significant association between peer quantity and prosocial behavior. Family functioning moderated the relationship between peer quality and prosocial behavior. ADF with higher quality peers are more likely to show more prosocial behavior, and poor family functioning would weaken the association between peer quality and prosocial behavior. The protection of ADF can begin by improving family functioning and guiding ADF to form relationships with high-quality peers.

Based on the World Health Organization’s (WHO) definition, adolescence is the phase of life between childhood and adulthood, from ages 10 to 19. Adolescence represents a developmental time window^1^. One of the characteristics of adolescence is that adolescents actively expand their social relationships beyond the family context. During this period, they have the highest need for social belonging, including popularity, group affiliation, and peer relationships^2^. While prosocial behavior is purely altruistic, it also emphasizes the individual’s performance and motivation for engaging in behavior that helps others. It is a continuum that ranges from self-benefit to benefiting others and holds greater personal meaning and social value. Prosociality is a multidimensional construct including proactive, reactive, and altruistic dimensions^3^. This study treats prosocial behavior as an altruistic dimension, where altruism’s motivation is in doing good for others, including modesty, helpfulness, cooperation, and sharing^4^. Prosocial behavior is important in developing mature social relationships and taking social responsibility^5,6^. Research revealed that adolescents who show prosocial behaviors are more liked by their peers and teachers^7^. Healthier friendships and teacher–student relationships enhance adolescents’ social belonging^8^. Additionally, studies have found that improved prosocial behaviors can increase adolescent academic achievement^9^.

Promoting health development for ADF in China is a relatively new topic, lacking extensive study. In 2013, the Ministry of Civil Affairs (MCA) issued the “Circular on the Pilot Work of Building an Adequate and Inclusive Child Welfare System” (Civil Letter [2013] No. 206)^10^, which provides an official definition of children (0–18 years old) from difficult families. Thus, MCA^10^ defined ADF as “(1) individuals whose parents are severely disabled or ill, (2) individuals whose parents are serving long-term sentences or in forced drug rehabilitation, (3) individuals whose one parent has died, and the other parent is unable to fulfill their child-rearing obligations and guardianship duties due to other circumstances, and (4) individuals from low-income families”. If any of these criteria apply, the individual qualifies as ADF. The MCA predicted ADFs would reach one million by 2022. ADF is a particularly vulnerable group. Due to their guardians’ parenting behaviors and the lack of proper care, these individuals are prone to social adaptation problems, resulting in poor prosocial behaviors^11,12^.

Prosocial behaviors are social behaviors that arise in the social environment. Therefore, we should study adolescents’ prosocial behaviors in the context of environmental factors—the peer environment is adolescents’ basic and essential environment^13^. Sullivan^14^ has demonstrated that peer presence promotes prosocial behavior among adolescents. A previous study has shown that young people with higher prosocial behaviors always had more friends than others^15^. Besides, young people select friends like themselves, including homogeneity in prosocial behavior^16^. Adolescents have enough time to observe and learn peers’ behaviors because they spend more time with them. Using two-year panel data, Busching^17^ found that adolescents could learn prosocial behavior from classroom peers. Thus, this study hypothesizes that more friends and high-quality peers are positively associated with prosocial behavior.

Enhancing our comprehension of the link between peer factors and prosocial behaviors requires studying possible moderating factors influencing this association. One such factor may be family functioning, which is the degree of closeness and care among family members, mutual support, and cooperation; it is closely related to individual emotions^18^. Young people with more supportive family environments may have more quality friendships, which can have a cumulative effect on mental health and behavior^19^. Less positive family functioning may exacerbate the potential adverse effects of peer factors on adolescent behavioral development^20^. Therefore, this study hypothesized that family functioning moderates the relationship between peer factors and prosocial behaviors.

Although the existing literature focuses on the influence of peer and family factors on adolescents’ prosocial behaviors, most studies focus on ordinary adolescents and lack exploration of vulnerable adolescents. For adolescents from difficult families, most studies have focused on the macro level, examining the establishment of a protection and welfare system. However, there is a lack of quantitative studies on prosocial behavior. This study explores the association between peer factors, family functioning, and prosocial behaviors among ADF. More specifically, the research aims to investigate (1) the direct association between peer factors (peer quality, peer quantity) and prosocial behaviors and (2) the moderation role of family functioning in the association between peer factors and prosocial behaviors. The results can help government policymakers and youth welfare departments better understand ADF’s current situation. We can then use this understanding to develop strategies, considering the perspectives of peers and families, to enhance adolescents’ prosocial behaviors. In the long run, focusing on adolescents’ prosocial behavior will promote healthy growth and foster societal harmony and stability.

## Methods

### Participants

This study used data from a major 2018 Chinese Ministry of Education project, “Research on the Health Status of Children from Difficult Families.” We conducted a field survey in August 2018 that lasted one month. We recorded the preparatory work details in our previous work^21^. The inclusion criteria of ADF relied on the official definition from the Chinese Ministry of Civil Affairs. We cooperated with local officials to randomly select ADF from the local registration dataset, which is up-to-date. Based on a stratified sampling method, we examined data from seven provinces. We randomly selected two provinces in China’s eastern, central, and western regions and one in the northeast, dividing the regions according to the National Bureau of Statistics standards. Next, we randomly selected three counties in each sample province, totaling 21 counties. Each district and county surveyed 100 children from four types of difficult families. The final sample size was 2,099 individuals (1,052 ages 6–10 and 1,047 ages 10–15). The response rate is 99%.

The study obtained informed consent from the participants’ guardians, and participants completed the questionnaire independently. However, the children’s guardians completed the questions about family conditions, such as income or parents’ education. After the participant answered, the investigator gave the participant a gift. In addition, relevant research bodies at the universities and local health departments provided ethical approval for this research. We conducted all methods following relevant guidelines and regulations.

In this study, we only included participants aged 10–15. This study investigates the relationship between peers, family functioning, and prosocial behaviors. However, considering children’s limited knowledge of family functioning and peer quality, we did not set questions about family functioning and peers for children aged 6–10. This study included 462 males and 585 females. Most adolescents' parents are still married (80%). 72.49% of ADF lived in brick-tiled bungalows, which are not comfortable living conditions.

### Measures

*Dependent variable.* Prosocial behavior was assessed with Chinese version of Goodman’s Strengths and Difficulties Questionnaire (SDQ)^22^. The Chinese version of this questionnaire has good reliability and validity^23^. The SDQ has five items describing children’s prosocial behavior. The scale scores each item from 1–3 *(1* = *not true; 2* = *somewhat true; 3* = *certainly true)*, with the scores of the five items summed to a continuous variable (total score = 5–15). The items are: (1) I try to be kind to others, and I care about their feelings, (2) I often share things with others (food, toys, pens), (3) I am always willing to help if someone is hurt, upset or unwell, (4) I am kind to those younger than me, and (5) I often volunteer to help others (parents, teachers, classmates). The Cronbach’s alpha coefficient for the scale was 0.716.

*Independent variables.* Peer factors covered two dimensions: peer quantity and peer quality. In terms of peer quantity, participants were asked, "How many good friends do you have?". The investigators did not provide guidance or a definition of what constitutes a good friend. It was left up to ADF to determine their own criteria for a good friend and to indicate the number of good friends they currently have.We measured the number of good friends as peer quantity. The scale for peer quality included nine items: truancy and absenteeism, violating school discipline, fighting, smoking and drinking, frequenting internet cafes and game halls, and dropped out of school. Adolescents answered, “Do your good friends have any of the following?” For each item, there are three options: *1* = *no, 2* = *one or two, and 3* = *a lot.* We summed these nine items. The Cronbach’s $\alpha$ coefficient for the scale was 0.717. The higher the score for the scale, the poorer the peer quality.

Family functioning was the moderator in this analysis. It was measured by the Chinese version of APGAR scale (Adaptability, Partnership, Growth, Affection, and Resolve) designed by Smilkstein in 1978^24^. The Chinese version of this scale has good reliability and validity^25^. This scale examines how individuals regard the relationships between family members. The scale includes five items: partnership, adaptability, growth, affection, and resolution. Each item has three possible responses, reflecting an individual’s satisfaction with each item, ranging from *0 (hardly ever)* to *2 (almost always).* The higher the total score, the poorer the family functioning. The Cronbach’s $\alpha$ coefficient for the scale is 0.813.

*Control variables.* Based on previous research on ADF, variables related to both prosocial behavior and peer factors were selected as control variables^11,12^. The covariates in this analysis included age, parents’ highest education level, family economic status. The age was a continuous variable. We measured the education level by the average education years *(0* = *illiterate, 5* = *primary school, 8* = *middle school, 11* = *high school, 14* = *junior college, 15* = *undergraduate college, 18* = *post-graduate*). We measured family economic status by the per capita annual household income (*RMB*).

### Analyses

We performed all the data analysis using Stata 15.0. The data followed a normal distribution. Firstly, descriptive analysis was performed using the mean and standard deviation. Additionally, the correlation between the variables was analyzed using the Pearson correlation coefficient. Secondly, hierarchical regression and subsequent simple slope analysis were conducted to examine the moderating effect of family functioning. Differences were considered statistically significant at *P* < 0.05.

### Ethics approval and consent to participate

The study protocol was approved by the Ethics Committee of School of Sociology and Population Studies, Renmin university of China. (Project identification code:17JJD840001). Informed consent was obtained from all participants and from their legal guardians (if participants are under 18), before the survey.

## Results

### Descriptive analysis and correlation analysis

Table 1 describes the basic information about ADF and compares the differences between four types of ADF. The average age of the ADF was 12 years old. In terms of parents' conditions, the average annual income is 9,047 RMB, and the parent with the highest level of education has an average of 10 years of education, equivalent to the first year of high school. The average score for prosocial behavior was 10.57, and the average score for peer quality was 12.39. ADF had an average of 4 good friends in the total sample. Besides, the average score of family functioning was 8.91. In the four types of ADF, the scores for prosocial behavior, peer quantity, peer quality, and family functioning were similar.

**Table 1:** Descriptive analysis.

	Total sample	Group 1	Group 2	Group 3	Group 4
Prosocial behavior	10.57 ± 2.34	10.56 ± 2.23	11.04 ± 2.51	10.19 ± 2.39	10.85 ± 2.25
Peer quantity	4.19 ± 3.03	3.97 ± 3.55	3.97 ± 1.81	4.04 ± 2.20	4.30 ± 3.52
Peer quality	12.39 ± 2.16	12.57 ± 2.24	12.89 ± 2.42	12.58 ± 2.12	12.25 ± 2.18
Family functioning	8.91 ± 2.90	9.37 ± 2.83	9.04 ± 3.00	9.47 ± 2.96	8.49 ± 2.78
Age	12.21 ± 1.45	12.38 ± 1.39	12.32 ± 1.46	12.25 ± 1.38	12.19 ± 1.50
Income	8.50 ± 1.08	8.25 ± 0.93	8.59 ± 0.96	8.49 ± 1.15	8.50 ± 1.02
Educational level	10.06 ± 3.90	9.85 ± 4.01	9.82 ± 4.10	10.30 ± 4.74	9.88 ± 3.14

Group 1: ADF whose parents are severely disabled or ill; Group 2: ADF whose parents are serving long-term sentences or in forced drug rehabilitation; Group 3: ADF whose one parent has died and the other parent is unable to fulfill their child-rearing obligations and guardianship duties due to other circumstances; Group 4: ADF from low-income families.

Table 2 showed the correlation analysis of each variable for ADF. We found a significant correlation between prosocial behavior and peer quantity, peer quality, family functioning, and age. Peer quantity significantly correlated with peer quality only. There was also a significant association between peer quality and family functioning, age, and income.

**Table 2:** Correlation analysis between variables.

	1	2	3	4	5	6	7
1. Prosocial behavior	–						
2. Peer quantity	0.138***	–					
3. Peer quality	− 0.203***	− 0.183***	–				
4. Family functioning	0.065*	− 0.059	0.096**	–			
5. Age	− 0.071*	0.052	0.068*	0.079*	–		
6. Income	− 0.001	− 0.009	− 0.067*	0.049	− 0.008	–	
7. Educational level	− 0.058	− 0.059	0.058	− 0.043	− 0.049	− 0.093**	–

Notes: *p<0.05; **p<0.01; ***p<0.001.

### Hierarchical regression analysis

Table 3 indicates the contributing factors of prosocial behavior among ADF. Model 1 incorporated only the control variables. The results indicated age, parents' education level were associated with prosocial behavior. Model 2 incorporated the independent variables of peer quantity and peer quality. Peer quantity was positively associated prosocial behavior. To interpret, ADF with more friends were more likely to show better prosocial behavior. Besides, there was a significantly negative association between peer quality and prosocial behavior. ADF with higher quality peers were more likely to show better prosocial behavior. Model 3 included the variable of family functioning as a moderator, as well as interaction terms (family functioning* peer quantity and family functioning* peer quality) based on model 2. The study found that peer quantity was no longer significantly associated with prosocial behavior. Peer quality remained significantly associated with prosocial behavior. In terms of moderating effect, the interaction variable "family functioning peer quality" was significantly associated with prosocial behavior. This indicated that family functioning significantly moderated the effect of peer quality on prosocial behavior.

**Table 3:** Hierarchical regression.

Variables	Model 1		Model 2		Model 3	
	$\beta$	$t$	$\beta$	$t$	$\beta$	$t$
Peer quantity			0.082***	3.45	− 0.110	− 1.52
Peer quality			− 0.191***	− 5.73	− 0.527***	− 5.02
Family functioning					− 0.497***	− 3.21
Peer quantity* Family functioning					0.026**	2.85
Peer quality* Family functioning					0.038**	3.35
Age	− 0.119*	− 2.38	− 0.107*	− 2.19	− 0.111*	− 2.29
Income	0.009	0.13	− 0.018	− 0.28	− 0.025	− 0.39
Educational Level	0.037*	2.00	0.026	1.46	0.027	1.50

Notes: *p<0.05; **p<0.01; ***p<0.001.

**Figure 1:**
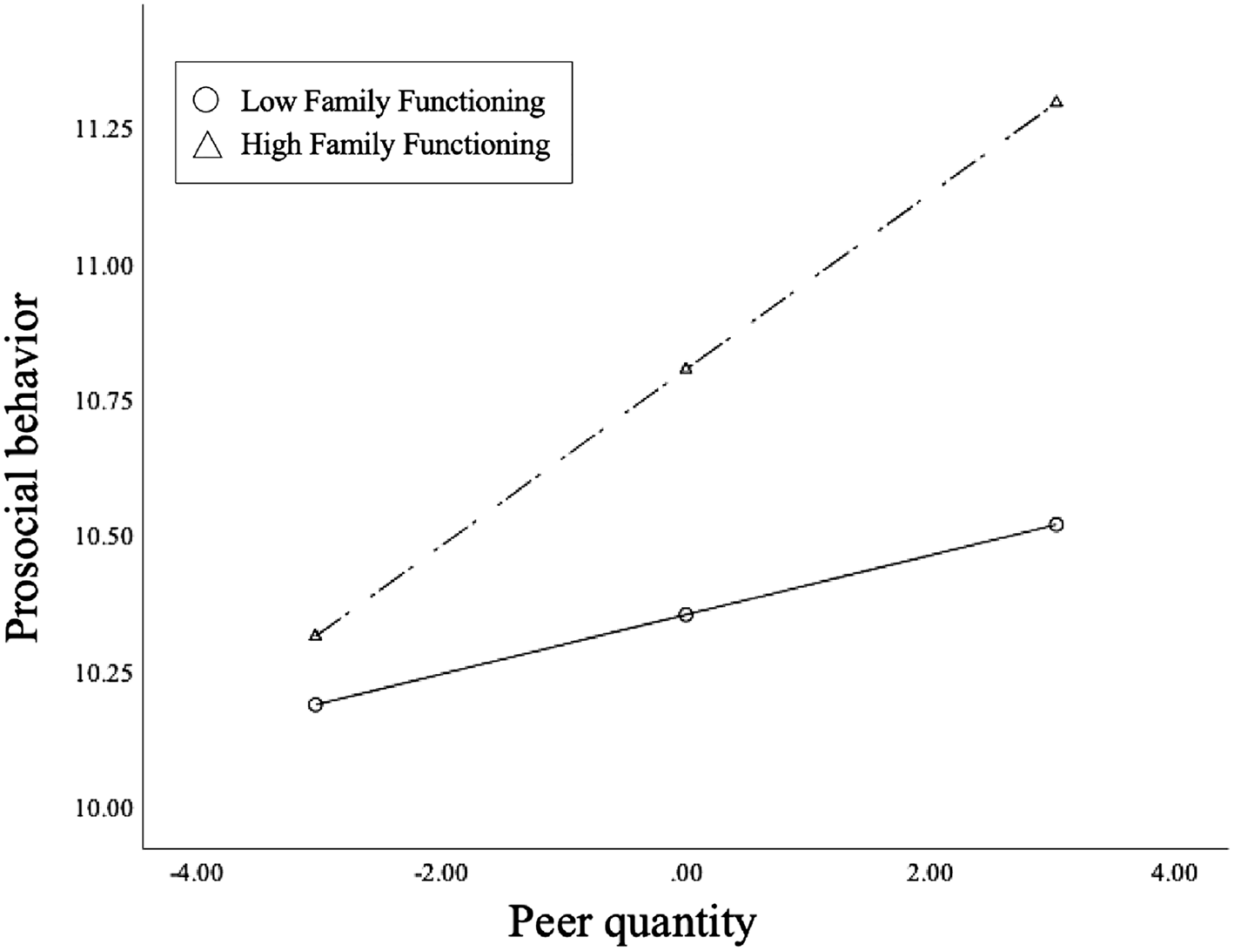
Simple slope analysis for prosocial behavior and peer quantity.

**Figure 2:**
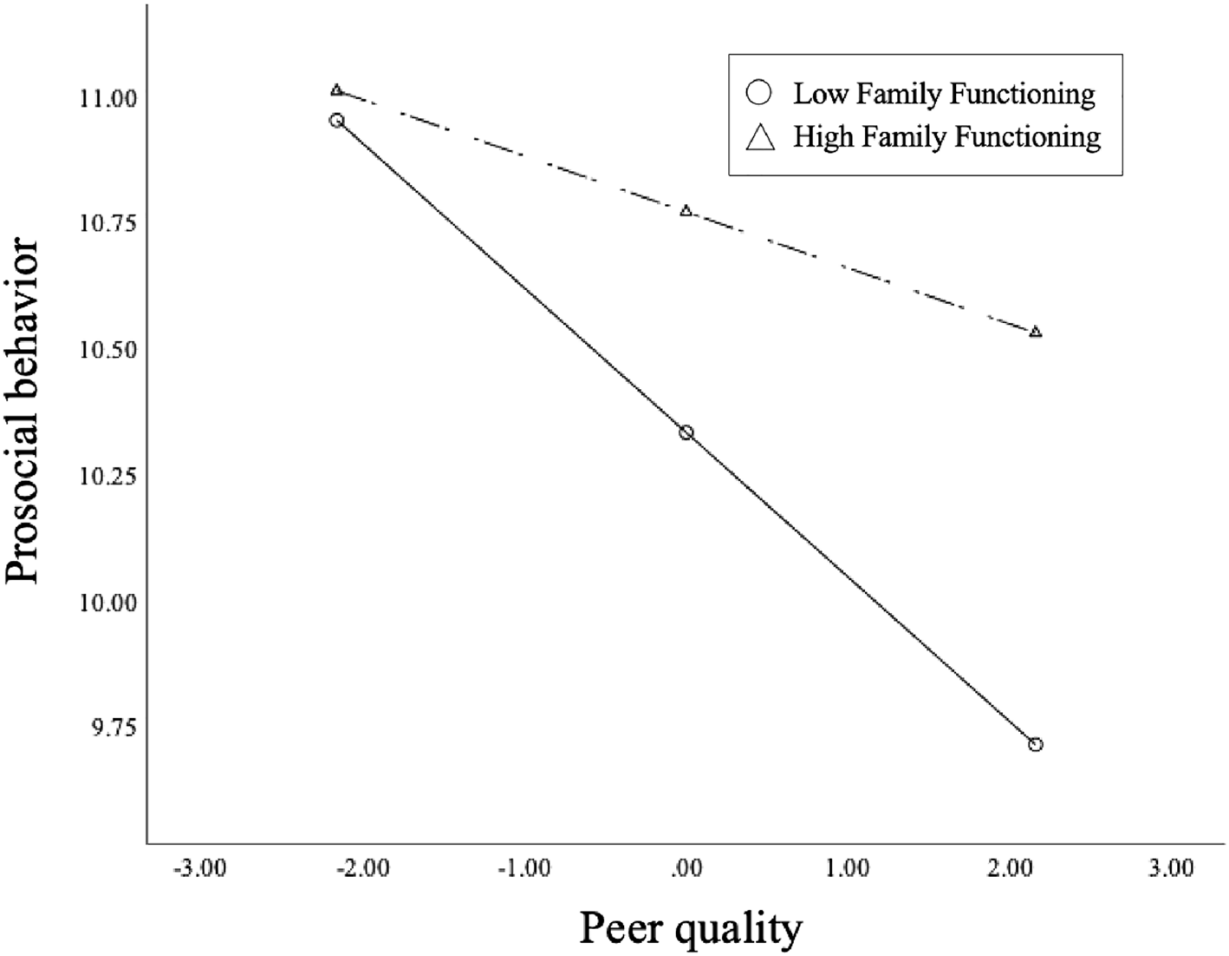
Simple slope analysis for prosocial behavior and peer quality.

For further analysis, we performed the simple slope analysis which can help understand “significant” interactions. With low level of family functioning, peer quantity was positively associated with prosocial behavior (${\beta }_{simple}$ = 0.05,$t$ = 1.98, $P$ = 0.048, 95% CI: 0.0006–0.1089). In Fig. 1, when comparing ADF with similar peer quantity, those who had better family functioning exhibited higher levels of prosocial behavior. This indicated that family functioning would strengthen the association between peer quantity and prosocial behavior. With low level of family functioning, peer quality was negatively associated with prosocial behavior (${\beta }_{simple}$= − 0.29, $t$ = − 6.26, $P$ =0.000, 95%CI: − 0.3765 ~  − 0.1968). In Fig. 2, when comparing ADF with similar peer quality, those who had lower family functioning exhibited lower levels of prosocial behavior. This indicated that family functioning would weaken the association between peer quality and prosocial behavior.

## Discussion

To our knowledge, this is the first study to investigate the association between peer factors, family functioning, and prosocial behavior among ADF. The study targeting ADF aims to help the public deepen their understanding of this special vulnerable group and promote improvements in the welfare system. This study aims to explore the direct relationship between peer factors and prosocial behavior, as well as the moderating role of family functioning in this relationship. The study collected data from 1047 participants as part of a major Chinese Ministry of Education project called "Research on the Health Status of Children from Difficult Families."

First, we found that peer quality was significantly associated with prosocial behavior among ADF. This means that adolescents who have higher-quality peers are more likely to exhibit prosocial behavior. This result was consistent with previous studies focus on adolescents from ordinary families and adolescents from low-income families^26,27^. According to social learning theory, peers play a modeling role in adolescent social interactions^28,29^. Near-peer role models, who are peers that are similar in age, gender, and social contact distance, tend to be respected, observed, and imitated by adolescents in terms of their behavior^30^. There was no significant association between peer quantity and prosocial behavior. There was limited research on the relationship between peer quantity and prosocial behavior. Previous study has showed that there are differences in prosocial behavior between adolescents with and without friends^31^. However, no studies have investigated the effect of the number of friends at its peak on prosocial behavior among adolescents. Combined with the quantity and quality of peers, we hypothesized that peer quality was more influential than peer quantity in shaping adolescents' prosocial behavior.

Second, this study found family functioning moderated the association between peer quality and prosocial behavior. Family and peer environment are all basic environment in the growth of adolescents^32^. Family functioning is an indicator that can comprehensively reflect the quality of the family environment. Some of the ADF's parents may migrate for work or be in prison, resulting in the separation of ADF from their parents for a long time or even being placed under the care of other guardians^33^. Thus, a warm family environment for ADF involves guardians, parents, siblings, and grandparents, etc. First, family functioning was associated with prosocial behavior. A warm family atmosphere provides a better psychological environment for the individual, motivating adolescents to engage in prosocial behavior such as sharing and helping^34^. Gao et al. has found the consistent results in left-behind adolescents^35^. Second, with improved family functioning, the emotional support for ADF transitions from low-quality peers to family members. For example, previous research has indicated that positive sibling relationships can help counteract the negative influence of peers^36^. As a result, ADF receive and internalize positive messages from their family members leading to a gradual increase in family influence on ADF. Furthermore, a warm family atmosphere provides a better psychological environment for the individual, motivating adolescents to engage in prosocial behavior such as sharing and helping^34^.

In terms of control variables, we found a significant association between age and prosocial behavior. It was consistent with previous studies. prosocial behaviors decreased with increasing age after entering adolescence^37^. This study used parents’ annual household income and educational level as measures of socioeconomic status, but no variables were associated with prosocial behaviors. This result is inconsistent with previous studies that examined socioeconomic status and prosocial behaviors^38^. Scholars suggested that children with higher socioeconomic status have more resources to help others, based on the family stress model^39^. Therefore, future studies should further examine the socioeconomic status variable.

Although this study provides the understanding about the relationship between peer factors, family functioning, and prosocial behavior among ADF, this study also has several limitations. First, this study used cross-sectional data. Therefore, we could not determine the causal relationship between peer factors and prosocial behavior among ADF. Future research needs to perform a follow-up study on adolescents from difficult families and analyze the influence of peers and family environment on prosocial behavior with longitudinal data. Second, we only collected the data from ADF, while it is important to show the difference between ADF and adolescents from ordinary families. Thus, future research needs to collect data from adolescent from ordinary families so that comparative analysis could be conducted.

## Conclusion

This study explored the relationship between peer factors, family functioning, and prosocial behavior among adolescents from difficult families. It was found that peer quality was significantly associated with prosocial behavior and family functioning performed a moderating role in the relationship between peer quality and prosocial behavior. However this study did not find a significant association between peer quantity and prosocial behavior. The results broaden the understanding of ADF. To promote prosocial behavior among ADF, peer relationships and creating a warm family environment are effective starting points. The welfare department could collaborate with the school to monitor the daily behaviors of ADF. Teachers can encourage ADF to make more friends and stay in touch with peers of good character. Furthermore, the community could also pay more attention to the family condition of ADF and provide timely support.

## Data Availability

Data requests will be considered on a case-by-case basis; please email the corresponding author.
